# Relevant Aspects of Centrifugation Step in the Preparation of Platelet-Rich Plasma

**DOI:** 10.1155/2014/176060

**Published:** 2014-03-25

**Authors:** Amanda G. M. Perez, José Fábio S. D. Lana, Ana Amélia Rodrigues, Angela Cristina M. Luzo, William D. Belangero, Maria Helena A. Santana

**Affiliations:** ^1^Department of Engineering of Materials and Bioprocesses, School of Chemical Engineering, University of Campinas, 13083-852 Campinas, SP, Brazil; ^2^Research Institute of Sports Medicine, Orthopedics and Regeneration, iMOR, 38050-400 Uberaba, MG, Brazil; ^3^Department of Orthopedics and Traumatology, Faculty of Medical Sciences, University of Campinas, 13083-887 Campinas, SP, Brazil; ^4^Haematology and Hemotherapy Center, Umbilical Cord Blood Bank, University of Campinas, 13083-970 Campinas, SP, Brazil

## Abstract

*Introduction*. Platelet-Rich Plasma (PRP) is rich in growth factors, playing important role in tissue healing. The wide variation of reported protocols for preparation of PRP leads to variable compositions, which induce different biological responses and prevent results comparison. This study aims to highlight relevant aspects of the centrifugation step to obtain reproducible results and overall quality. *Material and Methods*. Samples of blood were collected from 20 healthy donors that have signed free informed consent. Two centrifugation steps (spins) were analyzed for the influence of centrifugal acceleration, time, processed volume, and platelet gradient. The Pure Platelet-Rich Plasma (P-PRP) was characterized as platelet concentration, integrity, and viability (sP-selectin measurement). *Results*. Lower centrifugal accelerations favour platelet separation. The processing of 3.5 mL of blood at 100 ×g for 10 min (1st spin), 400 ×g for 10 min (2nd spin), withdrawing 2/3 of remnant plasma, promoted high platelet recovery (70–80%) and concentration (5x) maintaining platelet integrity and viability. The recovery of platelets was reduced for a larger WB volume (8.5 mL) processed. *Conclusion*. Centrifugal acceleration, time, WB processed volume, and minimization of the platelet gradient before sampling are relevant aspects to ensure reproducible compositions within the autologous nature of PRP.

## 1. Introduction

Platelet-Rich Plasma (PRP) is an autologous preparation that concentrates platelets in a small volume of plasma [1]. Platelets are rich in growth factors, which play an important role in tissue healing. Numerous studies have demonstrated the clinical application and notable results of PRP in dentistry [2], oral maxilla facial surgery [3], plastic surgery [4], orthopedics [5], rheumatology [6], and the treatment of different types of injuries that include chronic wounds [7, 8] and muscle injuries [9].

PRP is made for two purposes: one for harvesting platelets for therapeutic purposes and the other for testing for platelet function in PRP using aggregometry. In this work it was studied for therapeutic purposes only. 

**Figure 1:**
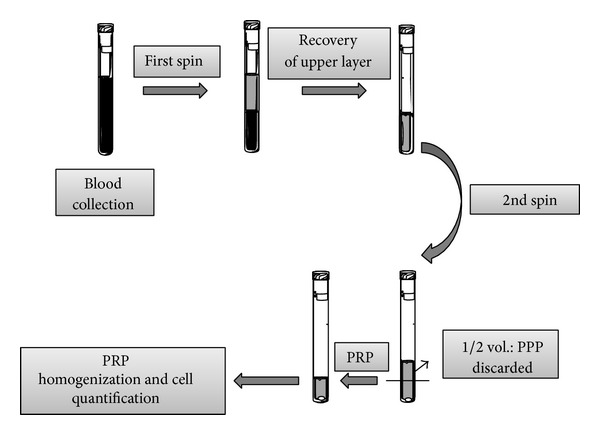
Flow chart describing the general preparation process of PRP. WB is initially collected in tubes that contain anticoagulants. The first spin step is performed at constant acceleration to separate RBCs from the remaining WB volume. After the first spin step, the WB separates into three layers: an upper layer that contains mostly platelets and WBC, an intermediate layer that is known as the buffy coat and that is rich in WBCs, and a bottom layer that consists mostly of RBCs. Only the upper layer or the upper layer plus buffy coat is transferred to an empty tube. The second spin step is then performed. The upper portion of the volume that is composed mostly of PPP (platelet-poor plasma) is removed to create the PRP (Platelet-Rich Plasma). The concentrations of platelets and WBC in each of the various layers are measured to characterize the quality of PRP.

The wide variation in the reported protocols for obtaining PRP may lead to samples with different compositions that may induce different biological responses [1]. Despite these variations, all protocols follow a generic sequence that consists of blood collection, an initial centrifugation to separate red blood cells (RBC), subsequent centrifugations to concentrate platelets, and other components and an activation of the sample by adding a platelet agonist (Figure 1). Prior to the platelet activation step, variables in the process that may influence the platelet integrity along with the composition and effectiveness of the PRP include the number of spins, centrifugal acceleration, and time period of centrifugation [10]. In addition to the platelets, the white blood cells (WBC) composition may also be analyzed, as the concentration of these cells is also an important factor in tissue healing [11].

There is currently disagreement in the literature over whether the presence of WBC in PRP provides any benefit. Proponents of PRP containing high WBC concentrations (Leukocyte and Platelet-Rich Plasma (L-PRP) according to Ehrenfest classification [12]) believe that the presence of WBC provides natural protection against infections and allergic responses [12, 13].

Other authors do not recommend the presence of high WBC concentration in PRP (Pure Platelet-Rich Plasma (P-PRP) according to Ehrenfest et al.) [12]. The presence of neutrophils, which are 65% of WBC and more than 95% of granulocytes, may be harmful because they destroy surrounding tissue, even if the tissue is not injured. These neutrophils release nonselective and toxic reactive oxygen species that include hypochlorite, superoxide, and hydroxyl radicals at high levels [14, 15]. Some studies have also shown that WBC concentration is directly correlated to catabolic gene expression in the tendon and ligaments. High catabolic gene expression may impair tissue healing [11, 16].

There are numerous protocols in the current literature that describe the optimal conditions for centrifugation. However, these various protocols have been optimized with respect to different variables of the process, such as volume of processed WB, sampling, number of spins, time period of centrifugation, and range of centrifugal acceleration. Considering the complexity of an autologous product such as PRP and the need for quality control in clinical applications, it is crucial to demonstrate process's ability to reproduce consistent results. Therefore, the purpose of this study is to highlight the relevant aspects involved in the centrifugation step of the PRP preparation and to discuss their implications for the final composition of the PRP. This paper is mainly focused on the PRP preparation without the use of commercial kits.

## 2. Materials and Methods

### 2.1. Blood Collection

This study was approved by the Ethics Committee of the Medical Sciences School of the University of Campinas (UNICAMP), CAAE: 0972.0.146.000-11.

In the preparation of P-PRP, a volume of approximately 3.15 mL of WB was collected in 3.5 mL tubes (Vacuette, Ref. 454327; Greiner Bio-One) that contained 0.35 mL of 3.2% sodium citrate, an anticoagulant. An 8.5 mL tube also was used for the comparison of the effects of the processed volume.

### 2.2. P-PRP Preparation

#### 2.2.1. First Spin

For the first spin, WB was centrifuged at different centrifugal forces ranging from 50 to 820 ×g (50, 70, 100, 190, 280, 370, 460, 550, and 820 ×g) for 10 min in a Routine 380 R centrifuge model (Hettich, Zentrifugen). After the formation of three layers (a bottom layer composed of RBC; an upper layer composed of plasma, platelets and some WBCs; and an intermediate layer, or buffy coat, composed mostly of WBCs), the upper layer was collected with a pipette. This collection was performed carefully to avoid disturbing the bottom layer of RBC and the buffy coat layer. Depending on the centrifugal force of the spin, the collected volume ranged from 1 to 2 mL. The collected sample was then transferred to an empty siliconized glass tube to be homogenized. After the sample was adequately mixed, a blood cell count was performed with cell counting equipment (Micros ES 60 Horiba).

#### 2.2.2. Second Spin

The WB was initially centrifuged at 100 ×g for 10 min for the first spin step. Approximately 1.2 mL of the upper layer of the sample that underwent the first spin step was collected and transferred to 6 empty tubes. The tubes were centrifuged again for 10 min at various centrifugal forces: 200, 400, 800, 1200, and 1600 ×g.

The upper half of the plasma volume, platelet poor plasma (PPP), was removed. The remaining volume of P-PRP was homogenized and analyzed for platelets and WBC. The P-PRP was characterized by (i) measuring the platelet concentration gradient prior to PPP removal; (ii) observing cell composition after the removal of PPP and subsequent sample mixing; and (iii) examining the integrity of the platelets.

### 2.3. P-PRP Characterization

#### 2.3.1. Composition after the First Spin

After the first spin step, the concentration of platelets and WBC in the upper layer was measured to calculate the recovery efficiencies of plasma (*E*_Pl_), platelets (*E*_Pt_), and WBC (*E*_WBC_) as well as the platelet concentration factor (Fc_Pt_). These values were calculated using (1).

WBC composition was also analyzed by measuring the concentration of lymphocytes, monocytes, and granulocytes.

Consider the following:

(1) EPl=Volume  of  upper  layer  Total  volume  of  WB×(1−hematocrit)×100EPt=NPt  (upper  layer)NPt  (WB)×100EWBC=NWBC  (upper  layer)NWBC  (WB)×100FcPt=CPt  (upper  layer)CPt  (WB),

where *N*_Pt  (upper  layer)_ and *N*_Pt  (WB)_ refer to the number of platelets in the respective upper layer and in WB (counts/(mm^3^) × 1000 × volumes in mL); *N*_WBC  (WB)_ and *N*_WBC  (upper  layer)_ refer to the number of WBC in the respective upper layer and in WB; and *Ct*_Pt  (upper  layer)_ and *Ct*_Pt  (WB)_ are the concentrations of platelets in the upper layer and in WB, respectively.

The effect of time of centrifugation on the first spin step was demonstrated by comparing the composition of platelets and WBC in the upper layer between 6 and 10 min periods of centrifugation at 100 ×g.

#### 2.3.2. Composition after the Second Spin

After the second spin step and the separation of PPP (2/3 or 1/2 of the upper volume) from the P-PRP sample, the recovery efficiencies of platelets and WBC, along with the platelet concentration factor, were calculated by (2) for both PPP and P-PRP volumes.

Consider the following:

(2) EPt  (PPP  or  P-PRP)=NPt  (PRP  or  P-PRP)NPt  (upper  layer)×100EWBC  (PPP  or  P-PRP)=NWBC  (PPP  or  P-PRP)NWBC  (upper  layer)×100FcPt=CPt  (P-PRP)CPt  (WB),

where *N*_Pt  (P-PRP  or  PPP)_ is the platelet number in P-PRP or PPP; *N*_WBC  (P-PRP  or  PPP)_ is the WBC number in P-PRP or PPP; and *C*_Pt  (P-PRP)_ is the concentration of platelets in P-PRP.

The effect of the volume of plasma centrifuged in the second spin step was quantified in terms of the recovery efficiency of platelets that remained in the PPP volume.

#### 2.3.3. Platelet Concentration Gradient

The platelet concentration gradient in the samples was analyzed immediately after the second spin step by positioning the needle of the hematological counter at various points along the tube, in order to find the end of the PPP and the beginning of the P-PRP. After the removal of the PPP (1/2 of the upper volume), the remaining P-PRP volume was analyzed with respect to platelet recovery. Before platelet counting, the P-PRP was mixed by inversion, manually for 30 seconds or mechanically for 30 and 60 min, for minimization of the gradient.

#### 2.3.4. Platelet Integrity

The influence of the centrifugal acceleration on the integrity of platelets in the P-PRP sample was evaluated by measuring the sP-selectin after the second spin step. Plasma sp-selectin reflects platelet activation and release of growth factors [17]. Therefore, in this case, integrity of platelets means they are not activated during centrifugation, and the range of initial concentration of sP-selectin in plasma is maintained.

All assays were performed using enzyme-linked immunosorbent assay (ELISA) kits (R&D Systems) according to the manufacturer's instructions and specifications.

#### 2.3.5. Processed Volume

The influence of the volume of WB processed was evaluated by using commercial tubes of 3.5 mL and 8.5 mL. The samples were centrifuged at 100 ×g and 10 min. Moreover, the effect of volume of PRP processed at the second spin was evaluated in tubes of 3.5 mL at 400 ×g and 10 min.

In all cases at least two experiments were performed.

## 3. Results

### 3.1. Experimental Aspects

#### 3.1.1. Effects of Time on Centrifugation

Table 1 compares the recovery efficiencies of plasma, platelets, and WBC in the upper layer after the first spin step. The first spin step was performed at 100 ×g for either 6 or 10 min. According to the physics of the centrifugation process, time and acceleration are the fundamental parameters that define the composition of the PRP sample after the first spin step. The effect of centrifugation time at a low spin setting was evaluated with respect to the concentration of WBC in the upper layer. Based on the data collected after the first spin step, it appears that longer time periods slightly increased platelet recovery and decreased the concentrations of WBC in the upper layer. Therefore, time could be a control parameter when low levels of WBC, such as granulocytes and lymphocytes, are required in the PRP sample. 

#### 3.1.2. Platelet Concentration Gradient

Platelet concentration gradients are formed after both spins. Various factors contribute to this gradient such as the sizes of platelets, from the range of peripheral platelets as measured in femtoliters (10^−15^ L), until the biological difference among individuals along with haematocrit variability. However, this gradient is more critical after the second spin step because some erythrocytes are inevitably present in the volume that was transferred from the first spin. The presence of these remaining RBC can generate a pellet at the bottom of the tube, which adsorb platelets and WBC on its surface, as evidenced experimentally. The manual mixing for a short period of time was insufficient to completely resuspend the platelets and large variability in platelet counting was observed. The samples mechanically mixed by tube inversion for 30 or 60 min had an improved recovery efficiency of platelets in P-PRP (75–80%) regardless of the centrifugal acceleration that was applied. Thus, approximately 20% of platelets remained adsorbed in the RBC pellet. According to Tukey's test (*P* = 0.05), there is no statistical differences for 30 or 60 min mixture. 

The residual of platelets in PPP was minimal when centrifugal accelerations of 400 ×g or higher were applied.

#### 3.1.3. Platelet Integrity

**Figure 2:**
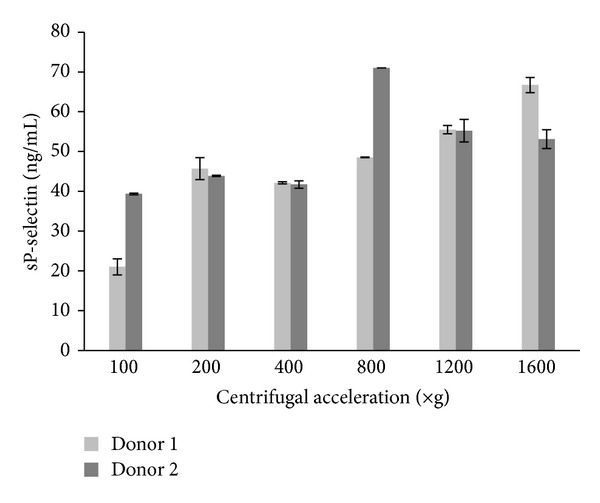
Effects of centrifugal acceleration on concentration of sP-selectin after the second spin.

The initial plasma concentrations of sP-selectin for two of the donors ranged from 18 to 40 ng/mL. These values are considered to be in the normal range of concentration values for human plasma [18].  Figure 2 shows that the concentration of sP-selectin from both donors effectively increased when the centrifugal acceleration was 800 ×g and 1200 ×g, indicating activation of the platelets during centrifugation (herein considered as loss of platelet integrity).

### 3.2. Performance of Centrifugation Step and P-PRP Composition in Two Spins

#### 3.2.1. First Spin

**Figure 3:**
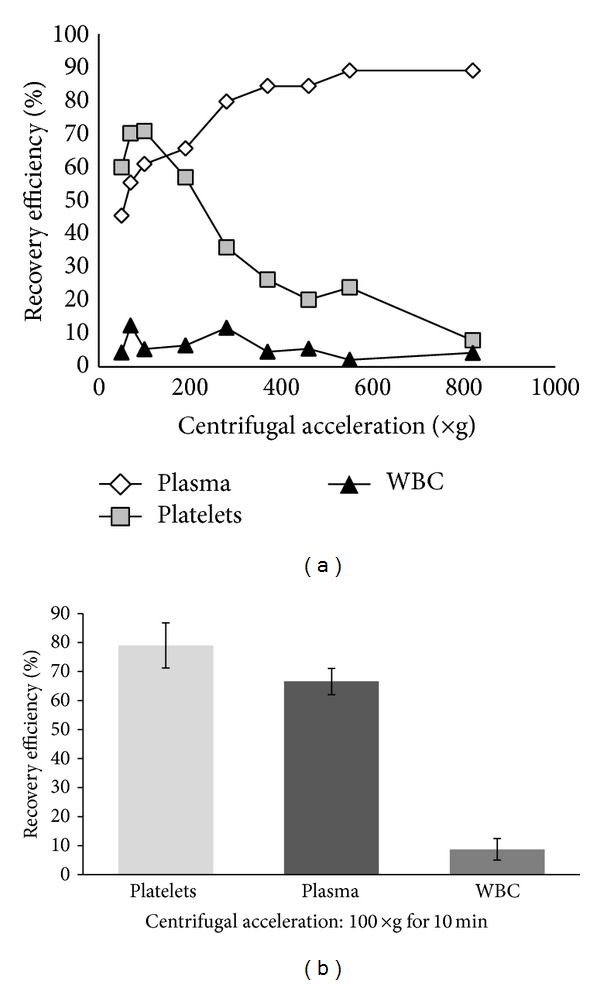
Recovery efficiency of platelets, plasma and WBC after the first spin step of WB: (a) centrifugal acceleration from 50 to 820 ×g for 10 min (*n* = 1); (b) centrifugal acceleration of 100 ×g for 10 min (*n* = 20).

The composition of P-PRP samples spun at various centrifugal accelerations is shown in Figure 3(a). The mean platelet count in WB for all donors was 250.0 Pq/mm^3^ (±45.2).

As expected, the recovery efficiency of platelets increased as the centrifugal acceleration increased from 50 to 70 ×g. The recovery efficiency peaked in the 70 to 100 ×g range and began to decrease in the 190 to 820 ×g range. The recovery efficiency of plasma increased with centrifugal force. The recovery of WBC in P-PRP remained between 5 and 10%, regardless of the applied centrifugal accelerations.

The setting of 100 ×g for centrifugal acceleration and time of 10 min was determined to yield the maximum recovery of platelets; these settings were repeated with 20 donors for data validation.  Figure 3(b) shows that the mean recovery of platelets, plasma, and WBC was approximately 80%, 65%, and 8%, respectively.

#### 3.2.2. Second Spin

After removal of the upper 1/2 of the plasma volume (PPP layer), the platelet concentration in the remaining P-PRP sample was approximately 3 times greater than the baseline concentration. To achieve a platelet concentration that was 5 times greater than baseline, it was necessary to remove 2/3 of the plasma volume after the second spin step (Table 2). For centrifugal acceleration ranging from 400 to 1600 ×g no WBC was measured in the PPP layer.

Despite the variable nature of PRP, it is possible to optimize the centrifugation process to produce PRP samples with consistent and reproducible compositions.

### 3.3. Effects of the Processed Volume

The processing of a larger volume of WB (8.5 mL) in a single tube decreased the recovery efficiency of platelets and the packing of the erythrocytes compared to 3.5 mL of WB processed at the same conditions. Therefore, in this situation, the centrifugal acceleration and time should be adjusted to achieve the same packing of erythrocytes and the recovery of platelets and plasma as a consequence.

Similar results were obtained when different volumes of the upper layer were transferred to the second spin. The concentration of platelets was favored and the percentage of remaining platelets in the PPP decreased for the smaller volumes processed.

## 4. Discussion

Centrifugation is one of the most widely used processes in liquid-liquid or solid-liquid separation. It is based on the application of a centrifugal force that is much higher than gravity. The difference in size and density of the particles in the various phases is the driving force responsible for the separation.

During the process of centrifugation, the movement of the particle is a result of the acting centrifugal force in the radial direction, the gravitational force in the downward direction, and the drag force in the opposing direction of particle motion. This frictional force is proportional to the particle's velocity and fluid viscosity in the Stokes regime of flow. All the mentioned forces are quickly balanced. The magnitude of the centrifugal force acting depends on the apparent mass of the particle (corrected to the buoyancy), the angular velocity, and its distance from the axis of the centrifugal head or rotor [19, 20]. The greater the distance from the rotor, the greater the centrifugal force acting on the particle.

In the case of WB, the centrifugal force and time drive the packing of erythrocytes at the bottom layer, the volume of plasma at the upper layer, and the recovery efficiency of platelets. According to the size of the commercial tubes assayed, the distances between the surface of WB and the rotor were 4.9 cm and 3.0 cm for the processed volumes 3.5 mL and 8.5 mL, respectively. Therefore for the same angular velocity, the mean centrifugal force applied on the erythrocytes decreased with the smaller mean distance from the rotor for the larger volume (8.5 mL) processed. These factors explain the decrease of the packing of erythrocyte at the bottom layer and also the recovery efficiency of platelets at the upper layer.

In order to restore the same separation efficiency, the packing of erythrocytes must be restored by an increase in time and centrifugal acceleration. According to the physical behavior, the extrapolation of the operating parameters is not straightforward, since it involves an exponential relationship with the distance traveled by the particles in the centrifugation.

This aspect is also relevant when different volumes are transferred from the first to the second spin. In this case, for the same parameters of centrifugation, the remaining amount of platelets in PPP varies due to the changes of the centrifugal force acting on the platelets. As a consequence, the platelet concentration factor is also altered.

After centrifugation of WB, a concentration gradient is formed within the tube for various blood components. Thus, to ensure reliable measurements, the sample should be previously well mixed. In the second spin step, the concentration gradients are more intense, because the platelets are adsorbing to the surface of the remaining erythrocytes. The presence of some RBC in the volume transferred from the first spin step is unavoidable. Therefore, an efficient mechanical agitation by inversion is required to ensure the platelet resuspension before concentration measurements.

Numerous protocols have attempted to optimize the centrifugation procedure using various performance standards and centrifugation parameters.

Anitua et al. [21] used only one centrifugation spin step and collected the volume immediately above the erythrocyte layer. This protocol obtained a platelet concentration factor of 2.67 above the baseline value. When all of the volume of the upper layer is collected, regardless of whether the buffy coat layer is included, additional spins can be performed to achieve higher platelet concentration factors (>3×) [22, 23].

Kahn et al. (1976) determined that a centrifugal acceleration of 3730 ×g for a period of 4 min was the optimal condition for obtaining the highest platelet concentration from 478 mL of WB [24]. The highest platelet recovery efficiency obtained by Slichter and Harker (1976) was 80%, using a sample of 250–450 mL of WB centrifuged at 1000 ×g for a period of 9 min [25]. It was observed that a subsequent centrifugation step of 3000 ×g for a period of 20 min decreased the platelet viability.

Landesberg et al. obtained PRP samples that had approximately 3.2 times the concentration of the WB baseline. The centrifugation procedure processed 5 mL of WB for two spins at 200 ×g for 10 min per spin [23]. In most of these studies, the authors only refer to the final concentration factor instead of recovery efficiency. Thus, the performance of theses protocols cannot be precisely measured. In our study, a maximum recovery of platelets (70–80%) was obtained from the first spin step when an acceleration of 100 ×g for 10 min was applied to 3.5 mL of WB (Figure 3). This processed sample had a concentration factor that was 2 times greater than the baseline concentration. After the second spin step, the platelet concentration factor of the PRP was 3 to 5 times greater than the baseline concentration by removing 1/2 or 1/3 of the upper volume.

In the same study, it was observed that centrifugal accelerations greater than 250 ×g resulted in a pellet of platelets that could not be resuspended [23]. In our study, it was shown that resuspension of approximately 85% of pellets was possible by mechanical mixing by inversion of the samples along 30 min. The resuspension of the platelets was successful even when high accelerations, such as 1600 ×g for 10 min, were applied.

Jo et al. achieved better efficiency (92%) by applying an acceleration of 900 ×g for 5 min for the first spin step [22]. A total of 9 mL of WB were processed, and the platelet concentration was measured to be 310.7 ± 78.5 × 10^3^/mm^3^. The maximum efficiency for the second spin step (84%) was obtained by applying 1500 ×g for 15 min. The platelet concentration was 633.2 ± 91.6 × 10^3^/mm^3^, which was 4.2 times greater than the baseline concentration. However, the integrity of platelets was not evaluated. Our results show that it is possible to achieve a concentration factor that is 5 times greater than baseline by applying a lower centrifugal acceleration in the second spin step (400 ×g) for less time (10 min). This setting ensures that platelets are not activated during centrifugation.

Bausset et al. [26] found that a centrifugation of 130 or 250 ×g for a period of 15 min was optimal when performing a procedure that involved two spins. A platelet concentration factor of 3.47 was obtained from the 8.5 mL WB processed, and 2.0 mL of plasma was processed in the second spin step. Although different methods were used, the authors evaluated the platelet integrity, and the data are consistent with those of our study.

Studying the first spin step, Araki et al. obtained a PRP sample that had 70–80% recovery of platelets and 10–35% recovery of WBC by applying low accelerations of 70 ×g for 10 min [27]. At 230–270 ×g, they achieved similar platelet recovery; however, the WBC recovery efficiency was only 4–6%. For the second spin step, an acceleration of 2300 ×g for 10 min was applied. The platelet concentration factor was 7.4 times greater than the baseline after removing approximately 1/10 of the PPP and adding ethylenediaminetetraacetic acid (EDTA) as an anticoagulant. However, when ACD was used, the platelet recovery efficiency was only 35%. The authors attributed this difference to the anticoagulant diminishing the platelet integrity in the sample. Therefore, the integrity of platelets is an important parameter that should be evaluated.

Mazzocca et al. [28] analyzed 3 protocols for preparing PRP samples with different compositions: a low platelet (382 × 10^3^/mm^3^) and low WBC (0.6 × 10^3^/mm^3^) process with one spin step at 1500 rpm for 5 min (10 mL WB); a high platelet (940 × 10^3^/mm^3^) and high WBC (17 × 10^3^/mm^3^) process with one spin step at 3200 rpm for 15 min (27 mL WB); and a double-spin process (1500 rpm for 5 min and 6300 rpm for 20 min) that produced a higher platelet concentration (472 × 10^3^/mm^3^) and lower WBC (1.5 × 10^3^/mm^3^). Many other studies [29, 30] specify centrifugal accelerations in rotations per minute (RPM) instead of in ×g, complicating the task of comparing and reproducing their results.

By following the highlighted aspects and the observations made in this study, it is possible to obtain the required composition of PRP, supported by reliable measurements and selected variables in the process. Efficient conditions for platelet recovery are low centrifugal acceleration (close to 100 ×g, 10 minutes) in the first spin and around 400 ×g in the second spin for preventing effects on activating platelets. Future applications of PRP preparations should also consider that the relative ratio of platelets to plasma proteins is disturbed.

## 5. Conclusions

Centrifugal acceleration, time, distance between the particles and the rotor to the volume of processed WB (or PRP in the second spin), prevention of platelet aggregation, and minimization of platelet gradient before measurements are the main relevant aspects to be controlled in the centrifugation step for the preparation and characterization of PRP. The observance of these aspects ensures the overall quality of PRP by allowing the variability of results to become restricted only to the autologous nature of the product. This is the starting point for comparison of biological results as well as for the standardization of the PRP for specific applications* in vivo*.

## Figures and Tables

**Table 1:** Comparison of the recovery efficiencies of plasma, platelets, and WBC in the upper layer after the first spin step of 100 ×g for 6 or 10 min. Volume of WB: 3.5 mL.

	Recovery efficiencies (%) (±SD)			WBC composition (±SD)		
	Plasma	Platelets	WBC	Lymphocytes (10^3^/mm^3^)	Monocytes (10^3^/mm^3^)	Granulocytes (10^3^/mm^3^)
Blood	—	—	—	1.72 ± 0.64	0.31 ± 0.14	4.01 ± 1.71
10 min	66.6 ± 4.5	79.0 ± 7.8	8.7 ±3.7	0.71 ± 0.34	0.062 ± 0.05	0.32 ± 0.15
6 min	43.9 ± 6.2	72.3 ± 2.9	26.6 ±11.4	3.18 ± 0.77	0.47 ± 0.34	1.20 ± 0.43

**Table 2:** Composition of platelets and WBCs in the P-PRP samples after the second spin step (400 ×g and 10 min). According to the hematocrit of the donor, volumes of the upper phase after first spin ranged from 1.0 to 1.4 mL.

	Fc_Pt_	Platelet × 10^3^/mm^3^ (±SD)	WBC × 10^3^/mm^3^ (±SD)
P-PRP after second spin step			
Blood	—	232 ± 28	—
1/2 of volume of PPP removed	3.1 ± 0.3	668 ± 34	2.2 ± 0.4
1/3 of volume of PPP removed	5.2 ± 0.5	1.222 ± 166	3.3 ± 0.4
